# A modERN resource: identification of *Drosophila* transcription factor candidate target genes using RNAi

**DOI:** 10.1093/genetics/iyad004

**Published:** 2023-01-19

**Authors:** William W Fisher, Ann S Hammonds, Richard Weiszmann, Benjamin W Booth, Louis Gevirtzman, Jaeda E J Patton, Connor A Kubo, Robert H Waterston, Susan E Celniker

**Affiliations:** Division of Biological Systems and Engineering, Lawrence Berkeley National Laboratory, Berkeley, CA 94720, USA; Division of Biological Systems and Engineering, Lawrence Berkeley National Laboratory, Berkeley, CA 94720, USA; Division of Biological Systems and Engineering, Lawrence Berkeley National Laboratory, Berkeley, CA 94720, USA; Division of Biological Systems and Engineering, Lawrence Berkeley National Laboratory, Berkeley, CA 94720, USA; Department of Genome Sciences, University of Washington School of Medicine, Seattle, WA 98195, USA; Department of Genome Sciences, University of Washington School of Medicine, Seattle, WA 98195, USA; Department of Genome Sciences, University of Washington School of Medicine, Seattle, WA 98195, USA; Department of Genome Sciences, University of Washington School of Medicine, Seattle, WA 98195, USA; Division of Biological Systems and Engineering, Lawrence Berkeley National Laboratory, Berkeley, CA 94720, USA

**Keywords:** *Drosophila*, transcription factors, regulation, embryo and gene expression

## Abstract

Transcription factors (TFs) play a key role in development and in cellular responses to the environment by activating or repressing the transcription of target genes in precise spatial and temporal patterns. In order to develop a catalog of target genes of *Drosophila melanogaster* TFs, the modERN consortium systematically knocked down the expression of TFs using RNAi in whole embryos followed by RNA-seq. We generated data for 45 TFs which have 18 different DNA-binding domains and are expressed in 15 of the 16 organ systems. The range of inactivation of the targeted TFs by RNAi ranged from log2fold change −3.52 to +0.49. The TFs also showed remarkable heterogeneity in the numbers of candidate target genes identified, with some generating thousands of candidates and others only tens. We present detailed analysis from five experiments, including those for three TFs that have been the focus of previous functional studies (*ERR*, *sens,* and *zfh2)* and two previously uncharacterized TFs (*sens-2* and *CG32006),* as well as short vignettes for selected additional experiments to illustrate the utility of this resource. The RNA-seq datasets are available through the ENCODE DCC (http://encodeproject.org) and the Sequence Read Archive (SRA). TF and target gene expression patterns can be found here: https://insitu.fruitfly.org. These studies provide data that facilitate scientific inquiries into the functions of individual TFs in key developmental, metabolic, defensive, and homeostatic regulatory pathways, as well as provide a broader perspective on how individual TFs work together in local networks during embryogenesis.

## Introduction

The *Drosophila melanogaster* genome is among the most thoroughly described metazoan genomes, a result of years of classical genetics studies followed by genome-wide efforts to identify transcripts and annotate DNA elements, particularly the modENCODE (Model Organism ENCyclopedia Of DNA Elements) (Brown and Celniker 2015) and modERN projects (Model Organism Encyclopedia of Regulatory Networks) (Kudron *et al.* 2018), http://epic.gs.washington.edu/modERN/). Classical genetic studies have identified major players in certain gene regulatory networks (GRNs), notably those TFs controlling early development (Nusslein-Volhard 1991; Wieschaus 2016). These features make *Drosophila* a powerful system in which to investigate transcription factor (TF) action at the genomic level.

Many of the TFs in flies have human orthologs, allowing the fly genes to be used to investigate the functions of these proteins during development (Lewis 1978). Studies on individual fly TFs have led to significant insights into the function of human disease genes specifically as well as human development and physiology more generally (Gehring 1996; Schott *et al*. 1998; Braun and Woollard 2009; Bellen *et al*. 2010; Kropp *et al*. 2019). Of the approximately 1,600 human TFs, only two-thirds have defined binding sites (Lambert *et al.* 2018) and one-third had detectable expression in the Human Tissue Atlas (Uhlen *et al.* 2015). Similar to the human TFs, nearly a third of *Drosophila* TFs remain largely unstudied and are known only by a curated gene identifier (CG) (Thurmond *et al.* 2019).

TFs play key roles in the complex GRNs that control development and physiology, including sex determination, early pattern formation, organogenesis, and response to environmental cues. TFs act by binding to specific DNA regulatory elements to control the expression of downstream genes. Sets of TFs often work collectively on adjacent or overlapping binding sites (Stanojevic *et al*. 1991; Li *et al*. 2008; Barr *et al*. 2017; Barr and Reinitz 2017) and often interact with one another. Catalogs of TF-binding regulatory sequences are underway in model genetic organisms and humans (Kudron *et al*. 2018; ENCODE Project Consortium et al. 2020), using chromatin immunoprecipitation sequencing assays (CHiP-seq) and other methods that detect physical interactions, such as yeast one-hybrid (Y1H) (Hens *et al*. 2011; Zhu *et al*. 2011) and two-hybrid (Y2H) (Shokri *et al.* 2019). Connecting physical binding to biological function can be challenging, however, as not all TF-binding appears to drive gene expression (Li *et al*. 2008; Fisher *et al*. 2012). Therefore, complementary approaches are needed to identify downstream target genes, validate predicted DNA regulatory regions, and, ultimately, to reconstruct GRNs. Transgenic cis-regulatory module (CRM) reporter studies have been used to identify predicted regulatory elements that are functional in vivo (Pfeiffer *et al*. 2008; Fisher *et al*. 2012; Kvon *et al*. 2014; Arbel *et al*. 2019). One complementary approach to identify downstream genes controlled by specific TFs is to knock out individual TFs and monitor changes in global gene expression. A convenient means of generating TF knockouts or knockdowns is RNA interference (RNAi), a post-transcriptional gene-silencing process using double-stranded RNAs (dsRNAs) homologous in sequence to the silenced genes (Fire et al. 1998; Hannon 2002).

We used RNAi to disrupt individual TF expression throughout embryonic development. The Transgenic RNAi Project (TRiP) (Perkins *et al*. 2015; Zirin *et al*. 2020) has generated a genome-scale collection of RNAi stocks that allow disruption of gene activity under UAS GAL4 control in the germ line or soma. These lines have been used with tissue-specific Gal4 drivers to restrict RNAi to particular tissues or developmental stages (Schnorrer *et al.* 2010). We used the TRiP short hairpin RNA (shRNA) lines in the Valium20 vector driven by a ubiquitous Gal4 driver to suppress the expression of *Drosophila* TF genes throughout embryogenesis and identify putative target genes by conducting whole embryo RNA-seq on the TF knockdown embryos. As Gal4-driven RNAi is most effective in post-blastoderm embryos, and as the blastoderm TF network has been studied extensively (reviewed in (Chipman 2020)), we focused on TFs with patterned expression post-blastoderm. A caveat with these experiments is the well-known issue of off-target effects (Meghana *et al.* 2006; Moffat *et al.* 2007). Such effects have been greatly reduced but not eliminated with the shRNA lines designed by the TRiP project (Ni *et al.* 2011).

Genome-scale identification of *Drosophila* TF candidate target genes will facilitate the generation of detailed network maps in this model organism, and help elucidate the role of orthologous genes in humans. Of the 9,732 embryonically expressed coding genes (RPKM ≥1), 3,597 (37%) are still CGs with little known about their function.

To demonstrate the utility of our RNAi approach, we present here a global analysis of the first 45 TF knockdown experiments in the ongoing modERN project along with more detailed results from five individual TF knockdown experiments. Three of the TFs (*ERR*, *sens,* and *zfh2)* have been the focus of previous functional studies and allow us to compare our results with other published analyses. We also include two less well-studied TFs: *sens-2,* for which targets had not previously been identified, and *CG32006,* whose orthologs have been primarily studied in other species. This study provides insights into the transcriptional regulatory circuits that control development.

## Methods

### Crosses for production of RNAi knockdown flies

TF knockdown was generated using RNAi crosses containing a shRNA construct from the TRiP collection (Perkins *et al*. 2015; Zirin *et al*. 2020); (https://fgr.hms.harvard.edu/fly-in-vivo-rnai), expressed in the Valium20 vector. The TF RNAi lines, drivers, and control RNAi are shown in Supplementary Table 47. To activate the shRNAi, the TRiP line males were crossed to females from a da-Gal4 driver line, BL95282 (formerly BL55849), which carries homozygous copies of the da-GAL4 driver on two chromosomes (w[*]; P{w[ + mW.hs] = GAL4-da.G32}2; P{w[ + mW.hs] = GAL4-da.G32}UH1) so that all of the F1 embryos have two copies of the da-Gal4 driver and one copy of the RNAi hairpin for shRNA constructs on either the second or third chromosome, depending on the location of the target gene. The double homozygous da-Gal4 driver stock is prone to breaking down, which is difficult to see phenotypically. Therefore, we recommend keeping several separate sublines. With each experimental set, we used the *CG32006* and *zfh2* TRiP lines as positive controls—the cross to da-Gal4 should be embryonic lethal. The crosses were set up in two standard culture bottles, each with approximately 100 homozygous RNAi males and 250 homozygous virgin females from the da-Gal 4 driver line. All crosses were maintained at 27°C to maximize the Gal4 function. (Note that subsequent experiments showed that *CG32006* and *zfh2* RNAi crosses were both embryonic lethal even when performed at 20°C, although no embryos were collected to generate RNA-Seq data). After 1–2 days, the crosses were transferred to minicages (Geneseesci.com, Cat#:59-101). Embryos were collected on molasses/agar plates in standard petri dishes that fit on the bottom of the cages. The flies were allowed to acclimate to the cages for 2 days before embryo collections began, and at least two 1-hour clearing collections immediately preceded the timed sample collections. For the 0–1.5 hours of embryo collections, eggs were processed directly after a 1.5 hours of laying period. For the peak expression period (specific to each TF) and 16–18 hours late collection, eggs from 2-hour collections were aged at 27°C to the appropriate age and then processed. Aged embryos were dechorionated in bleach (3% sodium hypochlorite) for 3 minutes, washed with deionized water, transferred to agarose blocks, gently blotted dry, and then transferred to tared microfuge tubes before flash freezing in liquid nitrogen and storing at −80°C.

### RNA isolation, library preparation, and sequencing

Frozen embryos were homogenized using the Pellet Pestle Cordless Motor (Kimble Cat. No. 749540-0000; Pellet Pestles Sigma Cat. No. Z359947). RNA was extracted from the homogenate using TRIzol Reagent (Thermo Fisher, Cat. No. 15596026), chloroform extraction, and isopropanol precipitation. RNA was resuspended in Nuclease Free Water (Ambion AM9930) and incubated over night before purification using the RNeasy Mini Kit (QIAGEN Cat. No. 74106). After Qiagen clean-up and quantification (NanoDrop ND1000v 3.5.2), 10-µl aliquots (300 ng/µl) were sent to the Waterston Lab at the University of Washington for library construction and sequencing.

Before library preparation, RNA sample integrity was assessed on an Agilent 2100 Bioanalyzer using the Agilent RNA 6000 Nano Kit (Agilent 5067-1511). Although we did not use qPCR to check for knockdown of the TF transcript this could be done before library construction as an additional control. Libraries for sequencing were then made using TruSeq Stranded mRNA Library Prep (llumina 20020594). Paired-end sequencing was performed on an Illumina NextSeq 550 machine using 150-cycle Illumina Nextseq 500/550 Kits v2.5 (Illumina 20024907) and standard Illumina primers.

### RNA-seq data processing pipeline

Raw FASTQ files were aligned to the *D. melanogaster* reference genome (Release 6) (Hoskins *et al.* 2015) using STAR aligner 2.7.3a (Dobin and Gingeras 2016) with default settings and up to 20 multiple alignments to produce BAM files. Subread featureCounts v2.0.1 was used to determine read counts overlapping genes. The HTSeq “htseq-count” command was run using the default “–nonunique none” option, which excludes reads overlapping multiple annotated gene regions. This setting can affect the downstream DESeq2 analysis by showing zero differential expression for multi-cistronic genes. If the differential expression for multi-cistronic genes is required, we recommend running “htseq-count” with “–nonunique fraction” or “–nonunique random” parameters, using our provided BAM alignment files, then running DESeq2 using the generated htseq-count files as inputs. Differential gene expression in the RNAi experimental samples compared to the mCherry controls was determined using DESeq2 1.28.0 (Love *et al.* 2014) using default parameters. DESeq2 outputs six parameters: base mean, log2FoldChange standard error, stat (*z* score), log2FoldChange values, and adjusted and nonadjusted *P*-values. The *P*-values in DESeq2 are calculated using the Benjamini and Hochberg method (Benjamini and Hochberg 1995).

### Gene ontology analysis

To classify proteins and determine enrichment we used the gene ontology (GO) Panther classification system (Gene Ontology Consortium 2021). For each TF in our study, we generated ranked lists based on log2fold change of the upregulated or downregulated target genes for each time window. We used the FBgn numbers to determine GO enrichment using the “GO Enrichment Analysis” tool at http://geneontology.org/ using the default Drosophila gene lists (Mi *et al.* 2019). The adjusted *P*-values (*P*_adj_) we report were determined using default parameters and the output is labeled “FDR” http://geneontology.org/. Gene enrichment results referenced in the paper, including annotation version and release date, are in Supplementary Table 48. Supplementary Table 49 lists gene symbols and corresponding gene names for all named genes. To identify genes regulated in common by multiple TFs, we used the open-source bioinformatics tool provided by MolBio Tools (http://www.molbiotools.com/).

### Quality control metrics

Fastp v0.20.1 (Chen *et al.* 2018) was used to check the sequencing read quality. Alignment statistics from STAR were used to ensure reasonable read coverage and mapping quality. We generated a clustered heatmap of the median of ratios normalized gene counts for all samples using a custom script based on R, DESeq2 (Love *et al*. 2014), and ggplot (https://ggplot2.tidyverse.org/reference/ggplot.html). The heatmap includes additional metadata such as batch date, timepoint, and TF that were useful for comparing sample read coverage.

In addition, we generate differential expression heatmaps for each experiment, with gene differential expression plotted against each sample timepoint. For these heatmaps, we use log2-fold change cutoff of less than or equal to −1 or greater than or equal to 1, with adjusted *P*-value cutoff less than or equal to 0.1.

We calculated *P*-values for the *zfh2* 12–14 hours rank order negative log2fold gene list using the cumulative distribution function (CDF) of the hypergeometric distribution (https://systems.crump.ucla.edu/hypergeometric/index.php).

## Results

### RNAi RNA-seq resource

To prioritize TFs for the TF RNAi RNA-seq experiments, we examined transcriptional profiles for each of the ∼700 TFs in the *Drosophila* genome using RNA-seq data from modENCODE and spatial expression data from the BDGP embryonic expression pattern database (Graveley *et al*. 2011; Hammonds *et al*. 2013), as well as functional information available from the literature. We prioritized TFs with patterned expression in post-blastoderm embryos and selected an embryonic stage when the factor has maximal expression or function. We performed RNAi RNA-seq using whole embryo preparations from three-time windows: the prezygotic developmental stages, before any expression of the zygotically expressed TF normally is observed (0–1.5 hours after egg lay [AEL], embryonic stages 0–3), from a 2-hour window centered on the period of peak expression, and from embryos 16–18 hours AEL (embryonic stages 16–17), to capture downstream effects of TF knockdown after most of the organ systems are well established. The period of peak expression is defined by the highest embryonic 2-hour RNA-seq window identified in the modENCODE transcriptional profiling embryonic dataset (Graveley *et al*. 2011). For two TFs, *Hr51* and *CG9876* we selected secondary peak stages because the normal expression exhibited two distinct expression peaks. In parallel experiments, we assessed whether the RNAi cross resulted in lethality or other phenotype at any stage of development.

**Fig. 1.**
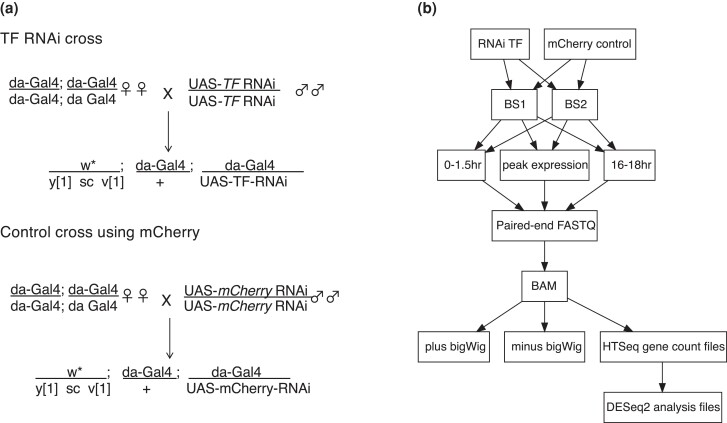
Cross scheme and experiment pipeline. a) cross schemes to generate RNAi knockdown embryos. Females carrying two homozygous copies of da-Gal4 were crossed to males homozygous for a specific TF RNA short hairpin loop under UAS to drive ubiquitous expression of the silencing hairpin. All of the progeny had two da-Gal4 transgenes driving expression of the TF RNA hairpin and one copy of the hairpin sequence. The scheme was carried out at 27C–27.5C. Embryos were collected after 2 hours egg lays and aged to the appropriate stage before chorions were removed and embryos frozen. b) The control cross activates the RNAi machinery against a target gene (*mCherry*) that does not exist in flies. Abbreviations: BS1 and BS2—Biosamples 1 and 2 (experiments done as replicates at three time points), BAM—Binary Alignment Map, bigwig (Binary wiggle tracks for viewing at UCSC, plus and minus refer to the DNA strands, HTSeq (High Throughput Sequence Analysis in Python), DESeq2 (Differential Gene Expression Analysis).

For these studies we crossed homozygous females from a ubiquitous Gal4 driver line to homozygous males from a UAS TRiP line to express short hairpin RNAs (shRNAs) and silence target TF expression via RNAi (Fig. 1a). TriP lines were chosen based on available homozygous Valium20 lines (containing 21 bp targeting sequence and vermillion gene for selection). Although the use of additional RNAi lines targeted at different sequences of each TF would help rule out off-target effects, we elected to use only one TRiP hairpin shRNAi for each TF because of two factors: the limitation of available lines (there is usually only one homozygous viable shRNAi line available for a given TF) and for the cost considerations in doubling the number of experiments. The Gal-4 driver gene is *daughterless* (*da),* which is ubiquitously expressed maternally and at all zygotic stages. The homozygous driver line carries da-Gal4 inserts on both the second and third chromosomes to maximize activation of the UAS regulated shRNA. We collected F1 embryos resulting from the cross and then isolated total RNA for analysis by RNA-seq to identify putative regulatory targets by changes in gene expression. For each experiment, two biological replicates were assayed for each time point along with two replicates of a control RNAi cross that targets mCherry (red fluorescent protein DsRed from *Discosoma sp*.), a gene not present in flies, so that the RNAi machinery is activated but without a target gene.

### Global analysis of RNAi dataset

We completed RNAi RNA-seq experiments (Fig. 1b) for 45 TFs studied in duplicate with at least three time points each, organized into 137 temporal datasets (399 files). The log2fold change in gene expression for each TF targeted by an shRNA during the period of peak expression and at 16–18 hours is shown in Table 1 and Supplementary Table 1. Fifteen TFs were knocked down with log2fold ≤−1.0 and another 15 with log2fold changes between −0.50 and −1.0. The remaining 15 TFs showed relatively low levels of RNAi inactivation, with log2fold changes no stronger than −0.48, including six with log2fold change no stronger than −0.15 at any of our measured time windows. We find that for TFs where RNAi knockdown resulted in lethality, the lethal period typically matched reported genetic studies of null mutations described below. This includes two members of the group with minimal to no knockdown, *sens-2* and *kay*; these will be discussed in more detail below. Of the 45 TFs, three were embryonic lethal (*caup*, *CG32006,* and *zfh2)*, nine were larval lethal (*CG10209*, *ERR*, *foxo*, *onecut*, *scro*, *sens, Sox15*, *trh*, and *Xbp1),* three were pupal lethal (*kay*, *sens-2,* and *TfAP-2)* and one, *Camta,* was larval/pupal lethal (Table 1). Lethality of *CG32006* and *sens-2* is reported for the first time here. The similarity of the lethal stages suggests these are not off-target effects and supports the idea that the RNAi may be acting to suppress translation as well as mRNA abundance. Alternatively, feedback mechanisms may lead to compensatory expression that does not fully restore function.

**Table 1.** Log2fold changes for each TF targeted by shRNAi at peak expression and 16–18 hours.

TF	Peak expression (hours)	Log2fold change peak	Log2fold change 16–18	RNAi Lethal Stage
*Bdp1*	6–8	−0.25	−0.19	—
*bsh*	10–12	−0.09	−0.01	—
*cad*	2–4	−0.60	−0.14	—
*Camta*	12–14	−0.55	−0.64	Larval/Pupal
*caup*	10–12	−3.52	−3.09	Embryonic
*CG10209*	12–14	−0.58	−1.02	Larval
*CG15696*	2–4	0.49	−0.91	—
*CG32006*	12–14	−2.35	−2.12	Embryonic
*CG33557*	6–8	−1.57	−0.45	—
*CG34376*	10–12	−0.05	−0.02	—
*CG9876* ^a^	6–8; 12–14	−0.31; −0.68	−1.58	—
*dac*	6–8	−0.52	−0.74	—
*dmrt99B*	6–8	−0.20	−0.42	—
*E5*	10–12	−0.15	0.16	—
*ERR*	10–12	−1.23	−1.14	Larval
*esn*	14–16	−0.86	−0.98	—
*Ets65A*	12–14	−0.65	−1.01	—
*fd59A*	10–12	−1.02	−1.44	—
*Fer1*	12–14	−0.40	−0.85	—
*foxo*	10–12	−0.39	−0.64	Larval
*gfzf*	2–4	0.19	0.01	—
*HLH54F*	8–10	−2.22	−1.50	—
*Hr3*	12–14	−0.48	−0.52	—
*Hr51* ^a^	6–8; 12–14	−0.53; −0.57	−0.47	—
*Kah*	8–10	−0.50	−0.51	—
*kay*	10–12	0.15	0.10	Pupal
*onecut*	12–14	−0.52	0.06	Larval
*pb*	8–10	−0.84	−0.48	—
*Pdp1*	14–16	−0.40	−0.29	—
*repo*	14–16	−0.58	−0.73	—
*scro*	12–14	0.00	−0.30	Larval
*scrt*	10–12	−1.03	−1.03	—
*sens*	6–8	−1.26	−0.90	Larval
*sens-2*	14–16	−0.09	0.06	Pupal
*Sox102F*	12–14	−0.32	−0.26	—
*Sox14*	6–8	−1.47	−0.74	—
*Sox15*	10–12	−0.71	−0.02	Larval
*ss*	12–14	−0.41	−0.49	—
*su(Hw)*	2–4	0.15	−0.75	—
*TfAP-2*	10–12	−0.26	−0.47	Pupal
*toe*	6–8	−1.80	−0.71	—
*trh*	6–8	−0.37	−0.43	Larval
*twi*	2–4	−1.11	−0.87	—
*Xbp1*	10–12	−2.78	−3.29	Larval
*zfh2*	12–14	−0.35	−0.27	Embryonic

Time point of 12–14 hours AEL was used in global analysis summary statistics.

**Fig. 2.**
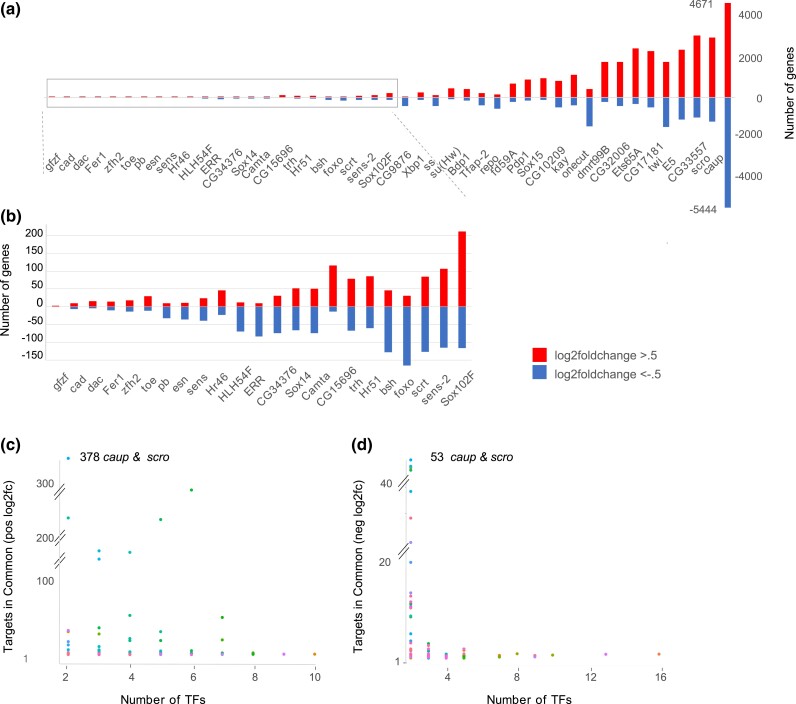
Log2fold change gene summaries. a) The number of genes with log2fold changes (≤−0.5 or ≥0.5) and *P*-value 0.1 at peak expression following knockdown of a single targeted TF in comparison to control embryos at the same stage for each of the 45 TF knockdown experiments. *X*-axis shows the names of the TFs subjected to RNAi; *Y*-axis shows the number of genes affected positively or negatively. b) Enlargement of the boxed area in (a) shows the smaller number of genes affected in these TF knockdowns. c, d) Combinations of target genes observed in common among different TF knockdowns at peak expression. The *X*-axis shows the number of TF experiments that share any targets. The *Y*-axis show the number of target genes in common. c) Genes upregulated in the TF knockdowns; d) Genes downregulated in the TF knockdowns. The colors used to represent the datapoints in (c) and (d) are randomly assigned by ggplot2 to distinguish overlapping points.

Since we found that RNAi knockdown shows a wide range in log2fold values for the TFs themselves, we determined the number of candidate target genes using log2fold cutoffs of less than −0.5 or more than +0.5 and a 0.1 adjusted *P*-value (*P*_adj_) (Fig. 2a, b). We found that although the knockdown of some TFs resulted in decreased or increased expression of only a few genes, knockdown of other TFs resulted in changed expression of hundreds or even thousands of genes (Fig. 2a, b). To evaluate whether the RNAi experiments identified unique sets of TF targets rather than a generalized RNAi response, we searched for target genes expressed in common (Fig. 2c, d). A few pairs of TF knockdown experiments with the highest number of affected genes (*e.g. Bdp1* and *caup*  Fig. 2c) shared hundreds of targets, but most TF pairs had few target genes in common, in either the positive (log2fold change ≥0.5) (Fig. 2c) or negative groups (log2fold change ≤−0.5) (Fig. 2d), indicating that the targets identified are largely distinct sets, specific to each TF.

After mapping reads to the reference genome and determining gene counts, we used the normalized read counts to produce a heatmap showing target gene clustering (Supplementary Figure 1). Clustering was done using R, DESeq2, and ggplot (custom script). As expected, we find that biological replicates clustered together. Similarly, RNA isolated from the same time points clustered together. The cluster diagram shows that the 0–1.5 hours samples clustered together, the 16–18 hours time points clustered together and the intermediate time points are relatively close to each other. At most, 20% of genes over the entire genome changed their expression as a consequence of a single TF being knocked down. In addition to the complete set of RNAi differential gene expression scores (Supplementary Table 1), likely candidate target genes with log2fold changes greater than 1.0 or less than −1.0 are listed in Supplementary Tables 2–46. Below we investigate the changes observed for five TFs, in each case briefly reviewing what was previously known for the gene, its expression pattern, and RNAi phenotype. Where known function might suggest targets, we relax the log2fold criteria to explore effects on these potential targets.

### *ERR* RNAi alters expression of genes involved in carbohydrate metabolism

The *Drosophila estrogen-related receptor* (*ERR)* is the single *Drosophila* member of the ERR subgroup from the nuclear receptor family of TFs, which acts through a conserved zinc finger DNA-binding domain and a C-terminal ligand-binding domain (Ostberg *et al.* 2003). Although the ligand for ERR is unknown, ERR has been found to regulate a mid-embryonic developmental switch that induces the expression of genes involved in carbohydrate metabolism, facilitating the dramatic growth of the larval stages (Tennessen *et al.* 2011). Studies in S2 cells and larvae found that ERR acts coordinately with the ecdysone receptor (EcR) (Kovalenko *et al*. 2019).

**Fig. 3.**
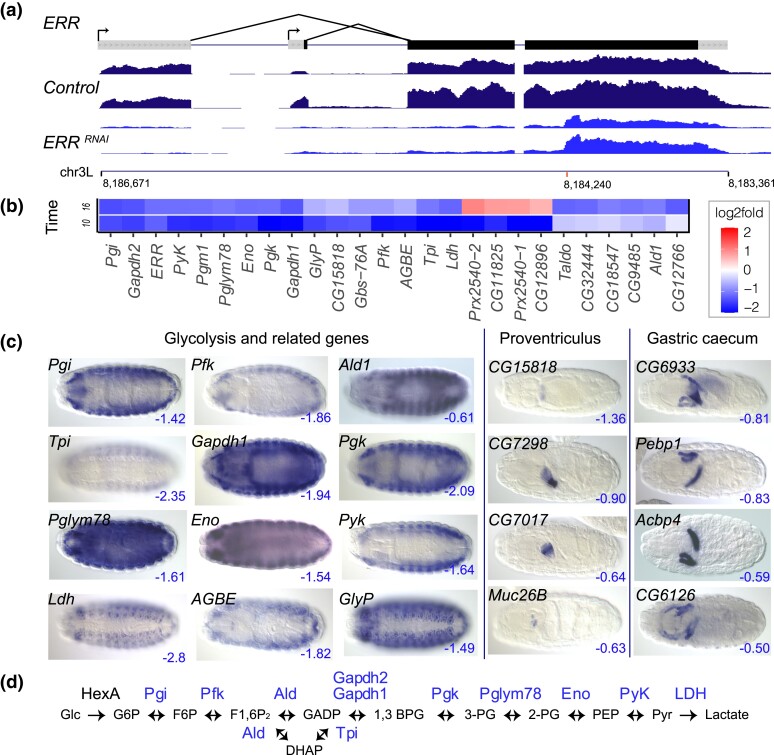
RNAi knockdown of *ERR*. a) Transcription unit of ERR is shown above RNA-seq tracks for the *mCherry* (control*)* and *ERR* RNAi knockdown embryos at 10–12 hours AEL. Grey boxes represent untranslated regions and black boxes coding exons. Each RNA-seq track is a single experiment, demonstrating experimental reproducibility. The *Y*-axis scale for the RNA-seq is 0–350 reads. Chromosome arm 3L coordinates are shown below the RNA-seq tracks, with a red mark indicating the position of the shRNA used for the RNAi experiment. b) The heatmap shows the genes with the most strongly reduced expression (blue, log2fold change ≤ −1) or strongly increased expression (red, log2fold change ≥1) in the *ERR* RNAi embryos at 10–12 hours (labeled “10”) and 16–18 hours (labeled “16”) AEL. A scale bar is shown on the right. C) RNA in situ expression patterns of ERR targets (log2fold change ≤ −0.5 blue) in wildtype embryos (Canton S). Embryo images are dorsal views with anterior to the left. Drosophila embryos range in length from 473–572 μm. d) Glycolysis pathway showing, in blue, proteins encoded by genes that are downregulated in the *ERR* RNAi embryos. *HexA,* the first gene in the pathway, showed a modest effect (log2fold change −0.28).

Peak expression for *ERR* is 10–12 hours AEL, when ERR is expressed ubiquitously in wild type embryos (Graveley *et al*. 2011; Hammonds *et al*. 2013). We found the *ERR* transcript itself had a log2fold change of −1.23 at 10–12 hours and −1.14 at 16–18 hours (Fig. 3a, Table 1 and Supplementary Table 16) and that the RNAi cross resulted in lethality at the larval stage, consistent with the null phenotype (Tennessen *et al.* 2011). We found 26 genes were downregulated with a log2fold change ≤−1.0 (and *P*_adj_ ≤ 0.1) at either 10–12 hours or 16–18 hours (Fig. 3b). Many of these genes are expressed in somatic muscle (Fig. 3c), including all *Drosophila* members of the aerobic glycolysis pathway except for *HexA,* the first gene in the biochemical pathway (Fig. 3d). Several more genes involved in carbohydrate metabolism were also reduced by at least log2fold −1.0, including *Gbs-76A* (regulation of glycogen biosynthetic process), *GlyP* (encoding glycogen phosphorylase), and *AGBE* (encoding 1,4-Alpha-Glucan Branching Enzyme*)* at 10–12 hours, and *CG12766* (encoding aldose reductase), *CG32444* (encoding aldose 1-epimerase*)*, *CG9485* (encoding 4-alpha-glucanotransferase), and *Transaldolase (Taldo),* at 16–18 hours. *Pgm1* (encoding phosphoglucomutase) was reduced by at least log2fold change −1.0 at both time points.

Because most genes are regulated by multiple TFs, the effects of expression loss of any single TF might be modest. Therefore, we reduced the stringency of our log2fold cutoffs to −0.50 to see if we could find potential target genes with shared expression patterns. Using GO enrichment analysis (http://geneontology.org/) and the list of *ERR* RNAi downregulated genes at 10–12 hours with log2fold change ≤ −0.5 and *P*_adj_ ≤ 0.05, we see an enrichment for genes with the Molecular Function GO Term “chitin binding,” (13-fold enrichment, *P*_adj_ 2.28E−05, see Supplementary Table 48). Five of these genes are expressed exclusively in the proventriculus during embryogenesis (*CG7298,* CG7017, *CG7714, Muc26B,* and *obst-J*), as is *CG15818,* with log2fold change −1.36 at 10–12 hours and the GO Term “carbohydrate binding”. Three genes are expressed primarily in the gastric caecum (*Pebp1*, *CG6126,* and the smORF *Acbp4*), while *CG6933* is expressed in both proventriculus and gastric caecum (Fig. 3c) and *Got2* (log2fold change −0.90) is expressed in both gastric caecum and somatic muscle. Additional investigations will be needed to validate these candidate targets. The log2fold changes for each of these genes was near zero at 16–18 hours, indicating that they may reflect processes that are active only in the earlier time window.

In addition, four genes, *CG12896, CG11825, Prx2540-1* and *Prx2540-2*, located within a 10-kb span of Chromosome II and which are involved in oxidoreductase activity, showed strongly decreased expression in 10–12 hours embryos (log2fold changes between −1.69 and −2.50) but increased expression at 16–18 hours (log2fold changes between +0.6 and 0.95, clearly visible in Fig. 3b). This could be a late embryonic ERR-regulated hypoxia response, similar to that reported by Li et al. (2013).

No other genes showed strongly increased expression (log2fold changes ≥1.0) in the ERR knockdown.

### *sens* RNAi alters expression of genes involved in chordotonal organs and ciliary assembly

The *senseless* (*sens*) gene encodes a Cys2–His2 (C2H2) zinc finger TF required for development of embryonic and adult peripheral nervous system. It is both necessary and sufficient for the development of sensory organs (Nolo *et al.* 2000). The zinc fingers of the Sens protein bind to specific DNA sites but also interact physically with bHLH proneural proteins, resulting in the dual function of *sens* as an activator or repressor depending on the levels of Sens protein relative to the levels of proneural proteins (Acar *et al*. 2006).

**Fig. 4.**
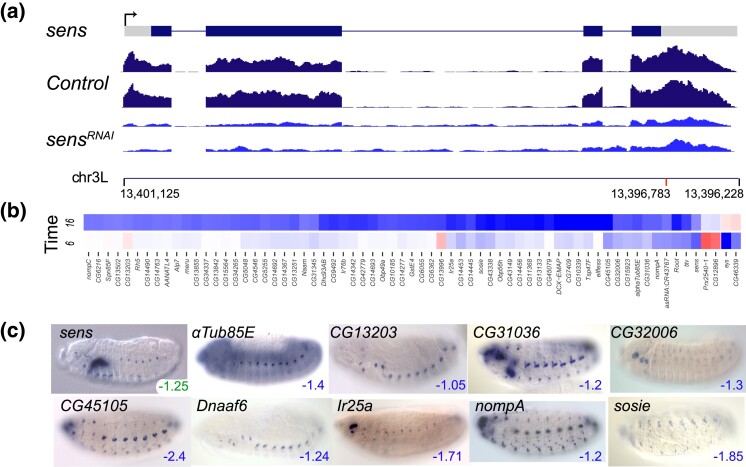
RNAi knockdown of *sens*. a) Transcription unit of *sens* is shown above RNA-seq tracks for the *mCherry* (control*)* and *sens* RNAi knockdown embryos at 6–8 hours AEL. The *Y*-axis scale for the RNA-seq is 0–100 reads. Grey boxes represent untranslated regions and black boxes coding exons. Chromosome arm 3L coordinates are shown below the RNA-seq tracks, with a red mark indicating the position of the shRNA used for the RNAi experiment. b) The heatmap shows the genes with the most strongly reduced expression (blue, log2fold change ≤ −1) in the *sens* RNAi embryos at 6–8 hours (labeled “6”) and 16–18 hours (labeled “16”) AEL. A scale bar is shown in Fig. 3b. Five of these genes have reduced expression at one time point (either 6–8 hours or 16–18 hours) and increased expression at the other (red). Genes with solely increased expression (log2fold change ≥1) are found in Supplementary Table 34. c) RNA expression patterns of *sens* targets in late-stage wild type embryos. Embryo images are dorsal views with anterior to the left. The log2fold scores for the pictured genes are indicated in the lower right corner of each image; maximum knockdown for *sens* is at (6–8 hours) AEL (green number) while all the target genes show maximum knockdown at 16–18 hours AEL (blue numbers). All show expression in the developing peripheral nervous system.

Peak expression for *sens* is 6–8 hours AEL, when *sens* is strongly expressed in primordia of the eye and antennae, in the sensory organs of the labial, labral, and maxillary sensory complexes, in dorsal, lateral, and ventral sensory complexes, and in salivary gland (Graveley *et al*. 2011; Hammonds *et al*. 2013). The *sens* transcript itself had a log2fold change of −1.26 at 6–8 hours and −0.90 at 16–18 hours (Fig. 4a). The RNAi cross resulted in lethality at the larval stage, consistent with the null phenotype (Nolo *et al.* 2000). The heatmap shows 67 genes that were downregulated with log2fold change ≤ −1.0 at either 6–8 hours or 16–18 hours (Fig. 4b). Of the 67 genes in the heatmap, 41 (61%) are annotated as computed genes (CGs) and have not yet been studied. From the RNAi candidate target gene set we searched for genes with embryonic gene expression patterns similar to that of *sens.* We identified nine genes that, based on their expression, may function in the visual primordia and peripheral nervous system, including chordotonal organs: *alphaTub85E*, *CG13203*, *CG31036, CG32006, CG45105*, *Dnaaf6, Ir25a, nompA,* and *sosie* (Fig. 4c). CG32006 is especially intriguing as it is a previously unstudied forkhead box TF that is also included in our study and is described below.

Nine of the 65 genes with log2fold change ≤ −1.0 at 6–8 hours or 16–18 hours have the Biological Process GO Term “sensory perception of sound” (29-fold enrichment, *P*_adj_ 5.15E−07, Supplementary Table 48): *btv, DCX-EMAP*, *Dhc1, Dhc93AB, nompC*, *Rh5,* and *Root,* as well as previously mentioned *sosie* and *nompA*. An additional five of the 65 genes do not yet have GO Terms but have been identified as being expressed in the adult fly hearing organ, known as Johnston's Organ: *CG13842, CG14342, CG14445, CG14693,* and *CG6362* (Senthilan *et al.* 2012). Looking for more subtle changes, we find 26 genes with log2fold changes between −0.50 and −1.0 which have either the GO Term “sensory perception of sound” (*Dnaaf4, Dnai2, iav, nan,* and *nompB)* or “Sensory Perception” (*Arr1, boss*, *btv, Crys, Ir21a*, *Ir25a, Ir76b, Obp47a, Obp49A, Obp50e, Obp56b, Obp56c, Obp56h, Obp58b, Obp58c, Obp58d, Orco, ppk, ppk26, tous,* and *SKIP)*.

Gene enrichment analysis further suggested that the 65 genes with log2fold change < −1.0 include 13 with the Cellular Component GO Term “cilium” (15-fold enrichment, *P*_adj_ 1.04E−08, Supplementary Table 48). One of these genes, *eys,* which is expressed in the scolopale space surrounding the cilium (Lee *et al.* 2008), had the strongest log2fold change in the 6–8 hours experiment (−2.17). Four other genes involved in cilium assembly had lesser log2fold changes at 6–8 hours of between −0.50 and −1.0 (*Cep89, CG3769, Ttc26* and *Ttc30)*. The remaining 12 cilium genes with log2fold changes ≤ −1.0 were all at 16–18 hours: *CG13251, CG13502, CG13855, CG14367, CG15923,* and seven genes grouped above with sensory perception of sound. Looking for more subtle effects, we found 23 additional cilia genes with log2fold changes between −0.50 and −1.0 at 16–18 hours (*Arl6, asl, BBS4, BBS8*, *BBS9, CG3085, CG7568, CG14020, CG15701, CG32668, CG45105*, *Cluap1, Cp110, Dnai2, dtr, Efhc1.2, nompB, Oseg2, Poc1, Rsph3, Tektin-C*, *TMEM216,* and *twy)* (Supplementary Table 1). Altogether, there were 33 downregulated genes with the Biological Process GO term “cilium organization” and log2fold values ≤ −0.5 (*P*_adj_ 3.90E−09, Supplementary Table 48). There were 49 genes with log2fold change ≥1.0, indicating increased expression (Supplementary Table 34), with 45 of those affected at 16–18 hours. There is no GO enrichment in this gene set.

### *zfh2* RNAi alters expression of genes in longitudinal glia

The *zinc finger homeodomain 2* (*zfh2*) gene has three homeodomains and sixteen C2H2 zinc fingers. It is expressed in the embryonic CNS and hindgut (Lai *et al*. 1991; Graveley *et al*. 2011; Hammonds *et al*. 2013) and has been shown to be specifically expressed in neuropile-associated glia and surface-associated glia (Beckervordersandforth *et al*. 2008). In larvae, *zfh2* is needed to establish proximo-distal boundaries in wing discs (Terriente *et al*. 2008) and leg discs and works with Notch to regulate apoptosis in leg discs (Guarner *et al*. 2014).

**Fig. 5.**
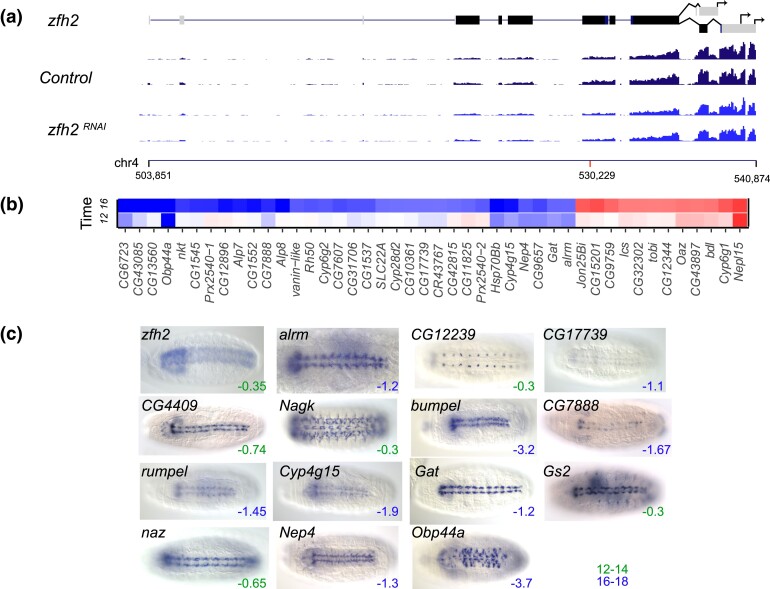
RNAi knockdown of *zhf2*. a) Transcription unit of *zfh2* is shown above RNA-seq tracks for the *mCherry* (control*)* and *zfh2* RNAi knockdown embryos at 12–14 hours AEL. Grey boxes represent untranslated regions and black boxes coding exons. The arrows represent alternate 3′ transcription termination sites. The *Y*-axis scale for the RNA-seq is 0–150 reads. Chromosome 4 coordinates are shown below the RNA-seq tracks, with a red mark indicating the position of the shRNA used for the RNAi experiment. b) The heatmap shows the genes with the most strongly reduced expression (blue, log2fold change ≤ −1) or strongly increased expression (red, log2fold change ≥1) in the *zfh2* RNAi embryos at 12–14 hours (labeled “12”) and 16–18 hours (labeled “16”) AEL. A scale bar is shown in Fig. 3b. c) RNA expression patterns of *zfh2* targets (log2fold change ≤ −0.25) in late-stage wild type embryos, showing expression in the developing CNS. Embryo images are dorsal views with anterior to the left. The log2fold scores for the pictured genes are indicated in the lower right corner of each image; green numbers indicate maximum effect at 12–14 hours AEL and blue numbers indicate maximum effect at 16–18 hours AEL.

Peak expression for *zfh2* is 12–14 hours AEL, when *zfh2* is first expressed in brain, longitudinal glia, and the hindgut of wild type embryos. In our *zfh2* RNAi experiments, the *zfh2* transcript itself had a log2fold change of just −0.35 at 12–14 hours and −0.27 at 16–18 hours (Fig. 5a) yet the RNAi cross resulted in 100% embryonic lethality, consistent with the null phenotype (Sun *et al.* 2004). The heatmap shows 44 genes, 31 of which had log2fold change ≤ −1.0 at 16–18 hours and one, *Obp44A*, with a log2fold change of −2.13 at 12–14 hours. *Obp44A* is expressed in late-stage embryos in longitudinal glia, ventral nerve cord, and brain (Fig. 5b).

In the 16–18 hours experiment, nine of the 31 genes with log2fold changes ≤ −1.0 are primarily expressed in longitudinal glia. In the 12–14 hours experiment, while only one gene, *Obp44a,* had a log2fold change of ≤ −1.0, 17 of the 30 genes with the most negative log2fold changes are expressed in longitudinal glia (https://www.fruitfly.org/). Nine of these had log2fold changes ≤ −0.5 (*alrm*, *bumpel*, *CG4409, Cyp4g15, Gat*, *naz, Nep4*, *rumpel*, and *wrapper*), and seven more had log2fold changes ≤ −0.3 (*CG7888*, *CG12239*, *Gs2*, *Jhbp*1, *Nagk*, *NimC4*, and *Csas)* (Fig. 5c).

To determine the significance of this enrichment, we calculated a *P*-value using the CDF of the hypergeometric distribution. We calculated a *P*-value of 2.9E−20 using the number of enriched genes, 17, in a sample size of 30 (rank order negative log2fold gene list at 12–14 hours), the total number of annotated longitudinal glial genes in *Drosophila*, 243, and the total number of embryonically expressed protein coding genes in *Drosophila,* 9732. Notably, the relatively low log2fold changes for many of these putative *zfh2* targets suggests that, for some genes, even small log2fold changes might indicate real effects of TF knockdown. Further investigations will be required to substantiate the role of *zfh2* in their regulation.

Four of the top nine downregulated target genes at 12–14 hours had the GO Molecular Function Term, “solute:sodium symporter activity.” The log2fold changes fall between −0.75 and −0.94 at 12–14 hours (>100-fold enrichment and *P*_adj_ 4.63E−05, Supplementary Table 48) and between −0.90 and −3.28 at 16–18 hours. Candidate genes *bumpel* and *rumpel* are both members of the solute carrier 5 family. *Gat* is a member of the solute carrier 6 family. *Eaat1* is a member of the solute carrier 1 family.

There were 12 genes with log2fold change ≥1.0, indicating increased expression in the knockdown embryos. Glia-expressed gene *Neprilysin-like 15 (Nepl15)* is the highest upregulated target in both the 12–14 hours and 16–18 experiments (log2fold changes of 1.62 and 1.51, respectively), indicating that Zfh2 may normally repress *Nepl15*. There are no statistically significant GO Terms in this small gene set.

### *sens-2* RNAi alters expression of genes involved in mannose metabolism

**Fig. 6.**
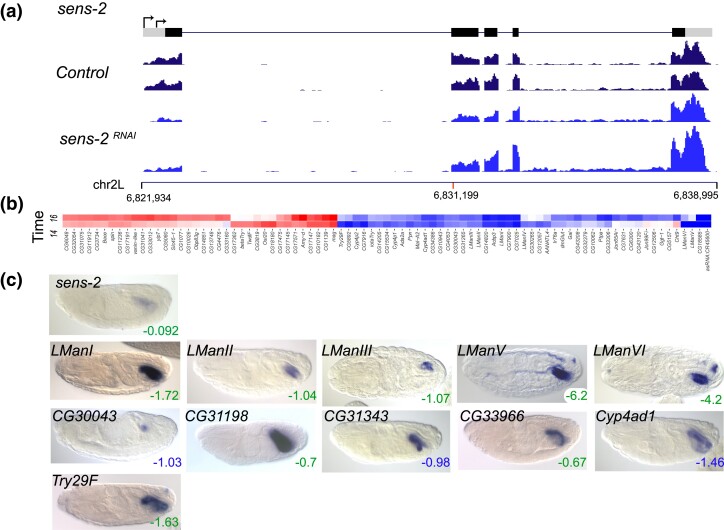
RNAi knockdown of *sens-2*. a) RNAi knockdown of *sens-2*. a) Transcription unit of *sens-2* is shown above RNA-seq tracks for the *mCherry* (control*)* and *sens-2* RNAi knockdown embryos at 14–16 hours AEL. Grey boxes represent untranslated regions and black boxes coding exons. The *Y*-axis scale for the RNA-seq is 0–150 reads. Chromosome arm 2L coordinates are shown below the RNA-seq tracks, with a red mark indicating the position of the shRNA used for the RNAi experiment. The RNA-seq shows reduced expression restricted to the region of the transcript 5′ of the shRNA site. b) The heatmap shows the genes with the most strongly reduced expression (blue, log2fold change ≤ −1) or strongly increased expression (red, log2fold change ≥1) in the *sens-2* RNAi embryos at 14–16 hours (labeled “14”) and 16–18 hours (labeled “16”) AEL. A scale bar is shown in Fig. 3b. c) RNA expression patterns of *sens-2* targets (log2fold change ≤ −0.50) in late-stage wild type embryos, showing specific expression in the fourth chamber of the midgut. Embryo images are dorsal views with anterior to the left. The log2fold scores for the pictured genes are indicated in the lower right corner of each image; green numbers indicate maximum effect at 14–16 hours AEL and blue numbers indicate maximum effect at 16–18 hours AEL.

The *senseless-2* (*sens-2)* gene encodes a C2H2 zinc finger TF with sequence similarity to the well-characterized *sens* gene (Jafar-Nejad and Bellen 2004). As described above, *sens* is expressed in the peripheral nervous system whereas *sens-2* is expressed in the fourth chamber of the late-stage embryonic midgut (Hammonds *et al.* 2013). The fourth chamber is the most metabolically active and immune responsive region of the gut. The biological function of *sens-2* has not been well studied. *sens-2* is first expressed in embryos starting at 4–6 hours AEL, with peak expression at 14–16 hours. In our *sens-2* RNAi experiments, the *sens-2* transcript itself had minimal log2fold change at both 14–16 hours (−0.09) and 16–18 hours (+0.06), yet the level of expression of the 5′ exon is reduced 2- to 3-fold (Fig. 6a). Although the log2fold change is minimal, the RNAi cross resulted in lethality at the pupal stage. The heatmap shows 49 genes that were downregulated with log2fold change ≤ −1.0 at either the peak expression period or at 16–18 hours (Fig. 6b). All six of the lysosomal class II alpha-mannosidases in *Drosophila* (*Lysosomal alpha-mannosidase I (LManI), LManII, LManIII, LManIV, LManV*, and *LManVI)* showed significantly reduced expression at 14–16 hours. *LManV* and *LManVI* had the two greatest negative log2fold changes in the genome (−6.19 and −4.26, respectively) while the remaining four alpha-mannosidases had negative log2fold changes between −1.04 and −1.72 (Molecular Function GO Term “alpha-mannosidase activity”, 200-fold enrichment, *P*_adj_ of 4.30E−09, Supplementary Table 48). Mannosidases are enzymes that remove mannose residues from glycoconjugates as part of glycoprotein degradation (Nemcovicova *et al.* 2013). These genes are expressed exclusively or primarily in the fourth chamber of the late-stage embryonic midgut, as is *sens-2* itself (Fig. 6c). Other genes expressed primarily in the fourth midgut chamber that showed reduced expression in the absence of *sens-2* include *Cyp4ad1, CG30043, CG31343, CG31198, CG33966* and *Try29f*, with log2fold changes between −0.67 and −1.63 at 14–16 hours.

There are 85 genes in the heatmap, 49 with log2fold changes ≤ −1.0 and 36 with log2fold changes ≥1.0 in at least one of the time windows, with 26 having the Molecular Function GO Term, “peptidase activity” (8.5 × enrichment, *P*_adj_ 3.64E−14, Supplementary Table 48). Fifteen such genes had decreased expression after *sens-2* RNAi while 11 had increased expression.

The regulation of these genes is likely modulated by interaction of *sens-2* with other known midgut expressed TFs (Buchon *et al.* 2013).

### *CG32006* RNAi alters expression of genes required for intraflagellar transport, including genes with human orthologs that cause Bardet-Biedl syndrome

*CG32006* was identified as a putative target of the TF *sens*, sharing an expression pattern in the peripheral nervous system and being knocked down by log2fold −1.30 in the *sens* 16–18 hours experiment. It encodes a protein with a forkhead box DNA-binding domain sequence of 80 to 100 amino acids. Its closest orthologs are Foxj1.2 in Xenopus, Foxj1b in zebrafish, and *fkh-8* in *C. elegans*. Foxj1 TFs had been identified as regulators of the production of motile cilia (Yu *et al.* 2008). The biological function of *CG32006* in Drosophila has not been studied previously.

**Fig. 7.**
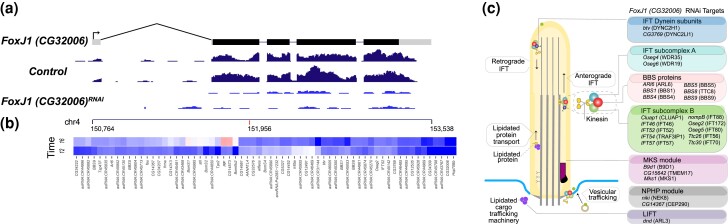
RNAi knockdown of *CG32006*. a) Transcription unit of *CG32006* is shown above RNA-seq tracks for two replicates each for the *mCherry* (control) and *CG32006* RNAi knockdown embryos at 12–14 hours AEL. Grey boxes represent untranslated regions and black boxes coding exons. *Y*-axis scale for the RNA-seq tracks is 0–50 reads. Chromosome 4 coordinates are shown below the RNA-seq tracks with a red mark indicating the position of the shRNA used for the RNAi experiment. b) The heatmap shows the genes with the most strongly reduced expression (blue, log2fold change ≤ −1) in *CG32006* RNAi embryos at 12–14 hours (labeled “12”) and 16–18 hours (labeled “16”) AEL. A scale bar is shown in Fig. 3b. Genes with increased expression (log2fold change ≥1) are in Supplementary Table 20. c) *CG32006* targets (log2foldchange ≤ −0.25) encoding functional components of ciliary trafficking (right, within colored boxes) shown with their positions within the ciliary trafficking system (left) (ciliary trafficking diagram (adapted from Reiter and Leroux 2017), and Adamiok-Ostrowska and Piekiełko-Witkowska 2020 (Adamiok-Ostrowska and Piekielko-Witkowska 2020)).

Peak expression for *CG32006* is 12–14 hours AEL, when it is expressed in the ventral and dorsal/lateral sensory complexes and in the sensory system of the head. In the *CG32006* RNAi experiments, the *CG32006* transcript itself had a log2fold change of −2.35 at 12–14 and −2.12 at 16–18 hours (Fig. 7a). The RNAi cross resulted in lethality at late embryonic stages. The heatmap shows 65 genes that are downregulated with log2fold change ≤ −1.0, with 45 downregulated at the peak expression period of 12−14 hours and 20 at 16–18 hours (Fig. 7b). Twenty-eight (40%) of these are antisense RNAs and one is a snoRNA. Of the remaining 36 genes, ten have the Biological Process GO Term “cilium assembly” (25-fold enrichment, *P*_adj_ of 5.45E−08, Supplementary Table 48) with half of these having orthologs that are implicated in the human ciliopathy disease known as Bardet-Biedl syndrome (BBS), a disease associated with mutations in the BBSome.

The BBSome is a protein complex that links signaling proteins to the intraflagellar transport machinery in cilia (Klink *et al.* 2020). In Drosophila, seven genes give rise to the BBSome (van Dam *et al.* 2013), five of which were knocked down with a log2fold change of at least −1.00 at one or both time points (*Arl6, BBS1, BBS5, BBS8* and *BBS9*), while BBS4 registered a log2fold change of −0.98 at 12–14 hours. In addition, 9 of 11 *Drosophila* genes of the Intraflagellar Transport Subcomplex-B (IFT-B), which governs anterograde transport, were downregulated in at least one timepoint with log2fold changes ≤ −0.5 (*Cluap1, IFT46, IFT52, IFT54*, *IFT57, nompB, Oseg2, Oseg5,* and *Ttc30*). Ttc26 had a log2fold change of −0.46 at 12–14 hours and −0.70 at 16–18. In addition, the IFT Subcomplex-A (IFT-A) gene *Oseg6* had a log2fold changes of −0.72 at 12–14 hours) (Fig. 7c).

Further supporting the role of CG32006 in cilia, several genes associated with two other ciliopathies, Meckel-Gruber syndrome (MKS) and nephronophthisis (NPHP), were also downregulated after CG32006 knockdown. MKS module genes *Mks1, B9d1* and *CG15642* were knocked down by log2fold changes −0.72 to −0.83, while NPHP module genes *CG14367* (ortholog of the human CFAP36 gene, an effector of ARL3) and *niki*, had log2fold changes of −1.1 and −0.88, respectively. The MKS and NPHP modules form a ciliary gate in the transition zone, helping to regulate passage of molecules into and out of the cilia (Williams *et al.* 2011).

In addition, we detected downregulation of the IFT Dynein subunit genes *btv* and *CG3769* (log2fold changes −0.97 and −1.02 at 12–14 hours). Finally, in further support of the role of *CG32006* in cilia, we reviewed other known ciliary genes and found evidence of down regulation, albeit at low levels for *CG45105* (ortholog of the human gene SDCCAG8, alias BBS16), found in the ciliary transition zone, and *dnd* (human ARL3), required for targeting proteins to the cilium (log2fold changes −0.47 and −0.30, respectively). There are 506 genes with log2fold changes ≥1.0, showing increased expression in the knockdown embryos, which is one of the larger repressed gene sets in our study.

### Findings from other RNAi experiments are consistent with known gene function or suggestive of previously unknown interactions

In addition to the five experiments highlighted above, other experiments generated intriguing target gene lists enriched for GO terms. Six of these are described below. They are involved in defense response *(kayak),* protein folding (*X box binding protein-1)*, neuron differentiation (*onecut* and *forkhead domain 59A)* and regulation of membrane potential *(scarecrow).* In addition, knockdown of *suppressor of Hairy wing* caused increased expression of antisense RNAs. These TFs are briefly described below.

*kayak* (*kay*) encodes a basic leucine zipper (bZIP) TF that, with the product from the TF *Jun-related antigen (Jra),* forms the Drosophila AP-1 heterodimeric TF complex (Perkins *et al*. 1990; Tran *et al*. 1998), required for stress and immune response as part of the Toll pathway (Valanne *et al*. 2011). *kay* is expressed in all embryonic stages, beginning with maternal deposition, with peak expression at 10–12 hours AEL, when *kay* is expressed in head mesoderm, amnioserosa, and midgut. Our *kay* RNAi experiment was pupal lethal in spite of small positive log2fold changes of 0.15 at 10–12 hours and 0.10 at 16–18 hours. In the 16–18 hours experiment, knockdown of *kay* upregulates 25 genes with the Biological Process GO Term “Defense Response” and log2fold change ≥ 1.0 (*P*_adj_ 4.36E−11, Supplementary Table 48), indicating that *kay* in embryos suppresses expression of these genes (Supplementary Tables 1 and 27). The gene set includes 10 of the 12 members of the Bomanin gene group, which make small peptides involved in immune response (Lindsay *et al.* 2018) as well as Defense Response genes *BaraA1, BaraA2*, *Bbd*, CG9372, *CG17738,* and *CG42259, Drs, Drsl2, Drsl4, Dso1, Dso2, GNBPlike-3, Listericin, LysS, Mtk, PGRP-LA, PGRP-SC1b,* and *SPH93*.

*X box binding protein-1(Xbp-1)* encodes a bZIP TF and is known to mediate the unfolded protein response (Souid *et al.* 2007). *Xbp-1* is spatially expressed in salivary gland, trachea, mesoderm, and hindgut (Hammonds *et al.* 2013). Our RNAi experiment was larval lethal, consistent with the results of mutant alleles of *Xbp1*, which die as second instar larvae (Buszczak *et al*. 2007). Knockdown of *Xbp-1* itself was very strong (log2fold −2.78 at 10–12 hours and −3.94 at 16–18 hours). There are 24 genes with log2fold change ≤ −1.0, of which eight have the Biological Process GO term “Protein Folding” (33-fold enrichment and *P*_adj_ 6.26E−07, Supplementary Table 48). (Supplementary Table 1). Looking at smaller changes in gene expression, Grp170 and Hsp27 (both “unfolded protein binding”) and CG11999 (“misfolded protein binding”), all had log2fold changes of ≤ −0.70.

*onecut* encodes a CUT homeodomain TF which by 8–10 hours AEL is expressed in the embryonic brain, ventral nerve cord, dorsal/lateral sensory complexes, and the stomatogastric nervous system (Hammonds *et al.* 2013). Mutant alleles are lethal (Boyle *et al.* 2006), consistent with our finding of larval lethality in the RNAi cross. Onecut is known to be important in the development and maintenance of neuromuscular junctions (Audouard *et al*. 2012). Our data shows strong enrichment for genes (Supplementary Tables 1 and 28) involved in the neuromuscular junction, particularly in the Neurexin Family Binding Protein genes *nlg1*, *nlg2*, *nlg3*, *nlg4*, *Nrx-1*, and *CASK*, which all had log2fold changes ≥ 0.5 indicating that they are normally downregulated by *onecut* at 16–18 hours. Other genes with positive log2fold changes ≥ 0.5 are enriched for the Biological Process GO term “neuron differentiation” (79 genes, 4.84E−12, Supplementary Table 48).

*forkhead domain 59A (fd59A)* is a relatively unstudied forkhead box TF. It is first expressed in a subset of brain cells at 6–8 hours AEL (stage 9) and at later stages is expressed in brain and ventral nerve cord. Existing alleles are viable, with decreased fecundity (Lacin *et al.* 2014). Our RNAi experiment was viable and knocks down *fd59A* by log2fold −1.0 at the peak expression window of 10–12 hours. Genes that are downregulated, albeit lowly (log2fold −0.40 to −0.71), by *fd59A* RNAi (Supplementary Tables 1 and 19) are enriched for the Biological Process GO term “neuron differentiation” (48 genes, 7.18E−08, Supplementary Table 48). Of the 48 neuron differentiation genes with reduced expression following knockdown of *fd59A*, 28 had increased expression following knockdown of *onecut* (Supplementary Table 48*)*.

*scarecrow (scro)* encodes a NK2 homeodomain containing protein and is expressed in the pharynx, the optic lobes and the ventral nerve cord (Hammonds *et al*. 2013). The TF *scro* activates 966 genes with log2fold changes of at least −0.5 at 12–14 hours (Supplementary Table 1). Of these, 27 have the Molecular Function GO term “regulation of membrane potential” (*P*_adj_ 1.17E−07), of which 12 map to an adult brain atlas (Davie *et al.* 2018) unannotated cluster, Cluster 13. Among the twelve are targets that include genes for nicotinic acetylcholine receptors *nAChRα, 1, 3, 5, 6,* and *7* and *nAChRβ1*, which encode acetylcholine-gated ion channels.

*suppressor of Hairy wing (su(Hw))* encodes a multifunctional zinc finger TF containing twelve zinc fingers (Parkhurst *et al.* 1988). First characterized for its insulator role (Roseman *et al.* 1993; Mallin *et al.* 1998; Soshnev *et al.* 2013), su(Hw) was later shown to have additional functions in direct transcriptional repression and activation. Genes with increased expression in the *su(Hw)* knockdown embryos are predominantly non-coding genes of the antisense class (110 of 181 or 61%) (Supplementary Table 40). They are distributed across all chromosomal arms and do not appear to be associated with any specific class of genes.

## Discussion

Of the 45 TFs profiled in our study, we surveyed 18 different DNA-binding domains. The five we focused on contain binding domains of the classes zf-C2H2 (two TFs), zf-C4 nuclear receptor (one), homeobox and zf-C2H2 (one), and forkhead (one), and are expressed in CNS, HindGut, Endoderm/Midgut, and PNS organ systems. DNA-binding motifs are known for sens, sens-2, and ERR. None have yet been determined for zfh2 or CG32006.

Cys2–His2 zinc finger proteins (ZFPs) are the largest group of TFs in higher metazoans (Enuameh *et al*. 2013) and HT selex (Nitta *et al.* 2015) has been used to identify in vitro binding domains in ZFPs.

The putative target lists of specific TFs identify genes of known and unknown function (genes with CG designations and genes without GO terms), providing an indication of the potential biological role of these uncharacterized genes. For instance, 61% of the genes in the *sens* heatmap are CGs. Several of these CGs have no GO terms, but a previous study provides supporting evidence that they are involved in hearing (Senthilan *et al.* 2012). We showed that another gene without GO Terms, *CG13203*, is expressed in the same sensory organs as the TF itself. It is likely that there are other putative targets about which little is known that are worth investigation based on differential expression levels shown in these studies.

These RNAi studies alone cannot distinguish between primary and secondary effects on target genes. Primary target genes trigger subsequent physiological events by acting on distinct biological pathways and modulating the expression of secondary target genes. For instance, we are unable to determine whether the chordotonal and ciliary genes that show reduced expression after *sens* knockdown are the result of direct interactions between Sens and the target gene sequence or are an effect of *sens* knockdown resulting in the loss of sensory precursor cells (Nolo *et al.* 2000). In either case, the data can be used to identify genes involved in these processes.

We observed variability in TF RNA self-knockdown, although the relationship between RNAi knockdown of TF RNA and subsequent expression of protein and downstream target RNAs is not yet well understood. Although we cannot rule out off-target effects, other data can be leveraged to support the observed RNAi results. For example, in the *sens-2* experiment, *sens-2* knockdown was minimal, yet two downregulated genes, *LManV* and *LManVI*, showed very large reductions in expression levels of −6.19 and −4.26, respectively. One possible explanation for this result might be that an off-target candidate gene is responsible for regulating *LManV* and *LManVI*; however, *sens-2* has a specific expression pattern in the fourth chamber of the midgut that is shared by all six alpha-mannosidase genes, as well as by several other genes with high differential expression (Fig. 6c). Since da-Gal4 is driving shRNAi expression in every cell at every stage, it seems unlikely that an off-target effect would cause reduced expression specifically in genes which share our targeted TF's expression pattern. It is also the case that we measured the log2fold change of *sens-2* only pre-blastoderm and at 14–18 hours AEL. It is possible that there is greater reduction in gene expression at earlier stages—it is first expressed 4–6 hours AEL—and that there is subsequent compensatory regulation.

Another variable in TF RNA knockdown that we cannot rule out for those TFs maternally expressed (15 of the 45) is that maternal proteins may mask the effects of RNAi knockdown. Of the three maternally expressed TFs we describe in detail, *ERR* perfectly recapitulates other work, while *kayak* gave intriguing gene knockdowns that need to be further investigated.

Although beyond the scope of this initial data release paper it will be valuable to more formally integrate these studies with other datasets such as ChIP, sc-seq, and many other functional genomics modalities. We expect that RNAi studies at single-cell resolution will show log2fold changes greater than those seen in whole embryo profiling, providing stronger separation of signal from noise and will in the future prove a useful addition to these whole embryo studies. In addition to improving signal to noise the single-cell studies will identify genes that are expressed across multiple cell types, and under the control of distinct regulatory modules and TFs.

Discussion of the specific five TF knockdowns follows:

### **ERR** (zf-C4)

ERR regulates the expression of the genes in the glycolytic pathway (Tennessen *et al*. 2011; Kovalenko *et al*. 2019; Beebe *et al*. 2020). Our data confirm this finding and validate the approach that whole embryo RNAi can replicate the findings of biochemical and S2 cell RNAi approaches. Although a complete study has yet to be done, we do find ERR-binding motifs (MAAGGTCA) (Nitta *et al.* 2015) in all genes of the pathway except for *HexA, Pfk,* and *Gapdh2,* suggesting direct binding of ERR to the genes of the glycolytic pathway. The Kovalenko study (Kovalenko *et al.* 2019) shows that EcR works with ERR to regulate glycolysis. We see EcR and ERR binding sites (Kudron *et al.* 2018) in close proximity in ChIP data near the glycolytic genes *Ldh* and *Gapdh1*. One question raised by Kovalenko *et al*. (2019) was the particular tissues that express both TFs. As the glycolytic pathway genes in embryos are expressed in somatic muscle it is likely the EcR-ERR functional interactions occur there.

### ***sens*** (zf-C2H2)

Chordotonal organs perform proprioceptive and other mechanosensory functions in invertebrates while stereocilia are the mechanosensing organelles in vertebrate animals, specifically in hair cells, which respond to fluid motion for various functions, including hearing and balance. Previous studies of *sens* indicated that *sens* has a dual role as a transcriptional repressor and activator, with low levels of *sens* repressing the transcription of the proneural bHLH gene *achaete*, and higher levels activating *achaete* transcription (Jafar-Nejad *et al*. 2003; Jafar-Nejad and Bellen 2004). Sens is also known to be regulated by *atonal* (Cachero *et al*. 2011) and in our study *ato* transcription is not affected by loss of *sens*. In the embryo, we find Sens plays an important role in regulating genes required for chordotonal organ function and ciliary assembly. This observation agrees with a genetic RNAi study that identified *sens*-depleted larvae as having abnormal chordotonal organs (Hassan *et al.* 2018). TFs identified in the Hassan study that are also downregulated in our RNAi study include *ss, cato, retn, Rfx, sv, insv* and the previously uncharacterized TF *CG32006,* providing an intriguing link between proneural genes and neuronal subtype differentiation.

### ***zfh2*** (Homeobox|zf-C2H2)

Previous studies of *zfh2* found that it regulates genes in a variety of tissues and in diverse processes including but not limited to: adult intestinal stem cells, where *zfh2* acts in parallel to insulin signaling and upstream of the TOR growth-promoting pathway (Rojas Villa *et al.* 2019); larval wing discs, where *zfh2* is required for specification of the proximal-distal domains (Terriente *et al.* 2008); and embryonic glial cells where *zfh2* was identified as being upregulated in over-expression studies of the TF *glial cells missing* (*gcm*) (Egger *et al.* 2002). Our data confirm the genetic misregulation studies and substantiate the role of *zfh2* in regulating genes expressed in embryonic glial cells. As no *zfh2* binding motif (Weirauch *et al.* 2014) has yet been described, it will be important to perform biochemical binding studies in the future. In reviewing single-cell studies of the adult brain (Davie *et al.* 2018) we find that 39 of the targets identified in our RNAi studies of embryos continue to be expressed in adult astrocytes (Cluster 10), ensheathing glia A and B (Clusters 14 and 35, respectively), chiasm glia (Cluster 82) and cortex glia (Cluster 60).

### ***sens-2*** (zf-C2H2)

*sens-2* was named for its sequence similarity to *sens* yet these genes have very different spatial expression patterns and appear to have very divergent functions. RNA-seq studies identified expression of *sens-2* in the larval and adult midgut (Graveley *et al.* 2011) and spatial expression in the embryo shows that *sens-2* localizes specifically to the posterior midgut (Hammonds *et al.* 2013). Consistent with these studies of the midgut are microarray experiments showing expression of sens-2 in R4 and R5, the most posterior divisions of the midgut (Buchon *et al.* 2013) and single-cell studies showing *sens-2* expression in midgut enterocytes (Hung *et al*. 2020). We show that Sens-2 regulates expression of mannosidases and more generally mediates genes required for specific digestive functions. Sens-2 shows strong homology to a human TF, growth factor independent 1B transcriptional repressor (GFI1B) (Hu *et al.* 2011). The human mannosidase proteins share sequence homology with the Drosophila mannosidase proteins. A rare but devastating human Lysosomal Storage Disease known as Alpha Mannosidosis is caused by mutations in the human gene MAN2B1, which is related to all of the lysosomal alpha-mannosidases knocked down by RNAi against *sens-2* in our experiments, with LManII being the closest ortholog. Drosophila has many models for various Lysosomal Storage Disorders (Rigon *et al.* 2021) but none yet for alpha mannosidosis. Our studies provide a hypothesis that GFI1B regulates MAN2B1, which could be tested in a fly model.

### *CG32006* (forkhead)

The identity of TFs that regulate genes involved in non-motile ciliary function, including those of the BBSome and IFT-A and IFT-B complexes, are not completely known. Our studies suggest a new unstudied forkhead domain TF, *CG32006*, is a key regulator. Transcription of *CG32006* is likely controlled, at least in part, by the zf-C2H2 TF, *sens,* which is a direct target of atonal, an HLH TF and one of the key proneural genes in Drosophila (Cachero *et al.* 2011). No other TFs associated with chordotonal development are affected by the *CG32006* RNAi, suggesting they are upstream in the developmental hierarchy.

The human genome encodes 50 forkhead box genes divided into 19 subfamilies (reviewed in (Jackson *et al.* 2010)). Based on amino acid sequence similarity searches (Hu *et al.* 2011) there are nine potential human orthologs of CG32006. FOXJ1 is the ortholog most closely associated with the regulation of cilia development (Brekman et al. 2014; Mukherjee *et al*. 2019) and Brekman *et al.* were able to rescue a phenotype of shortened cilia by overexpressing FOXJ1 in human tissue culture cells that had been treated with cigarette smoke extract. It seems highly likely that CG32006 is the fly ortholog of FOXJ1 and we suggest that it should be renamed FoxJ1.

Future directions for studies of the remaining 650 Drosophila TFs should be prioritized in two ways: (1) those showing strong RNAi phenotypes that match independently reproducible phenotypes and (2) those uncharacterized TFs with human orthologs, especially those associated with human diseases. Some TFs like B-H1 and B-H2 may have redundant functions. For these types of TF pairs reducing expression of each independently and both together will be required. In addition, for genes like *Jra*, whose product forms a heterodimer with *kay*, reducing expression of each independently will allow us to determine if candidate target lists are similar. It will be interesting to explore these and other heterodimer TF pairs both independently and in combination.

## Supplementary Material

iyad004_Supplementary_Data

## Data Availability

Strains are available from the Bloomington Drosophila Stock Center (BDSC) public repository, and identifying information is given in Supplementary Table 47. RNA-seq datasets and all metadata are available at http://encodeproject.org and the SRA. Users accessing the DCC ENCODE site can directly enter a TF of interest into the search bar in the upper right-hand corner and then choose the RNAi RNA-seq experiment from the data types listed. The experiment summary page provides all necessary information for the TF and the RNA-seq experiment, such as the strain genotype, library and sequencing platform information, and associated images, documents and files. All accession and identifying information for each dataset is listed in Supplementary Table 50. Supplementary Figure 1 and Table 1 are available in figshare: https://doi.org/10.25386/genetics.21585612. Supplemental material available at GENETICS online.
