# Supplementary material for: A modERN resource: identification of Drosophila transcription factor candidate target genes using RNAi
Source: Genetics. 2023 Jan 19;223(4):iyad004. doi: 10.1093/genetics/iyad004 (PMC10078917; doi:10.1093/genetics/iyad004)

RNAi Plot Expression w/ Covariates 2022−04−26.17−23−17

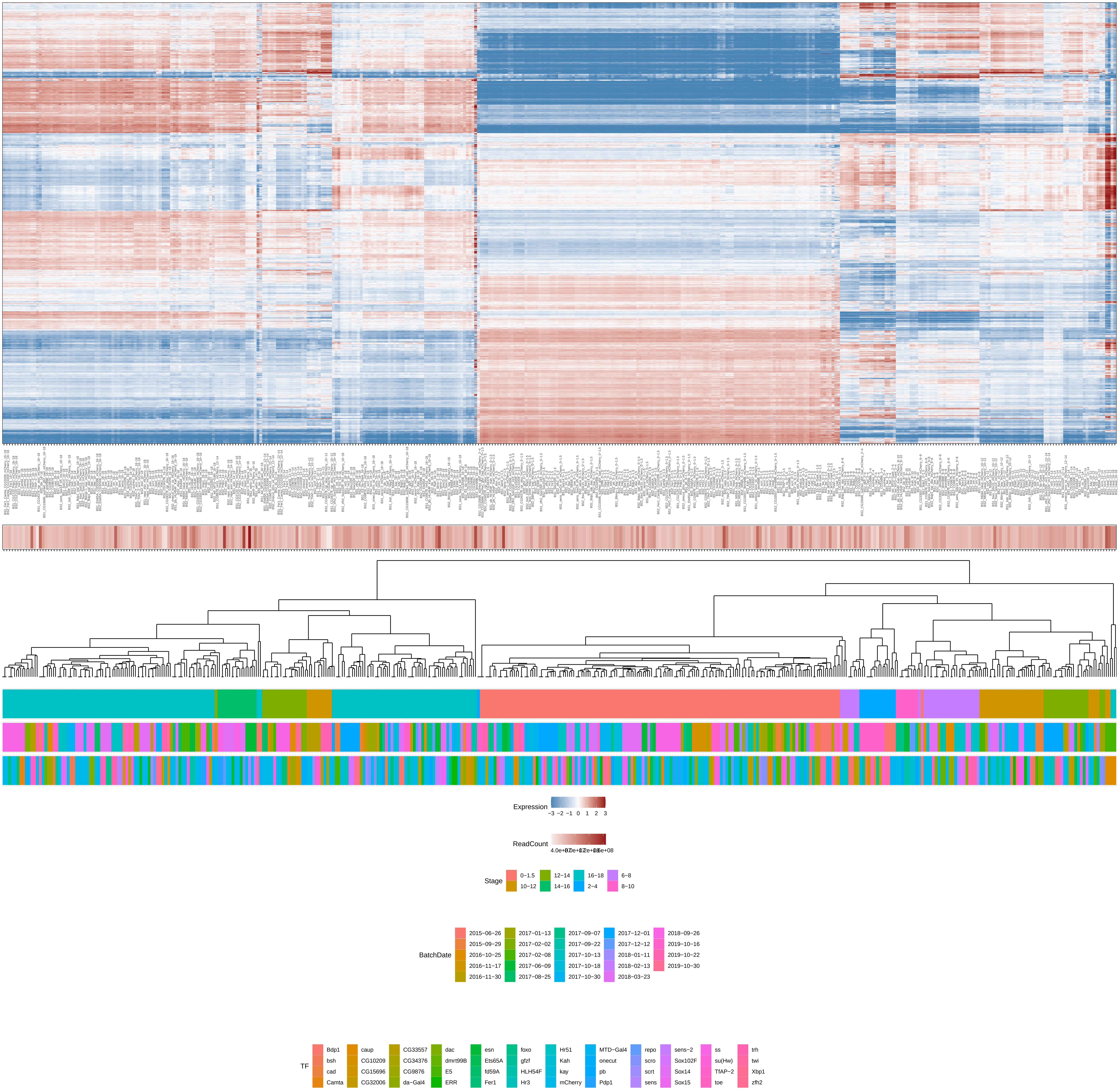

Supplement: iyad004_Supplementary_Data [file modERN Resource RNAi_Figure S1.RNAi plot expression with covariates.6-17-2022.pdf › modERN Resource RNAi_Figure S1.RNAi plot expression with covariates.6-17-2022.pdf]
